# IDR searcher: a search engine solution for public image resources

**DOI:** 10.1093/bioinformatics/btag486

**Published:** 2026-07-01

**Authors:** Khaled Mohamed, William Moore, Dominik Lindner, Josh Moore, Jason R Swedlow, Petr Walczysko, Frances Wong, Jean-Marie Burel

**Affiliations:** Faculty of Life Sciences, University of Dundee, Dundee, United Kingdom; Faculty of Life Sciences, University of Dundee, Dundee, United Kingdom; Faculty of Life Sciences, University of Dundee, Dundee, United Kingdom; German BioImaging, Gesellschaft für Mikroskopie und Bildanalyse e. V, Konstanz, Germany; Faculty of Life Sciences, University of Dundee, Dundee, United Kingdom; Biohub, Redwood City, CA United States; Faculty of Life Sciences, University of Dundee, Dundee, United Kingdom; Faculty of Life Sciences, University of Dundee, Dundee, United Kingdom; Faculty of Life Sciences, University of Dundee, Dundee, United Kingdom

## Abstract

**Motivation:**

In recent years, public image resources have emerged, but finding quality data efficiently remains a challenge, therefore limiting reuse.

**Results:**

IDR searcher is an open-source search engine designed to facilitate the exploration of datasets hosted in public bioimaging resources. The application offers a fast, efficient, cost-effective solution for discovering datasets and has the potential to address current disparities in finding quality datasets for exploratory research and can be combined with metadata visualization tools to enhance usability for the scientific community.

**Availability:**

IDR searcher is deployed using Ansible playbooks and released under the GPL v2 license. The source code associated with this manuscript is available at https://doi.org/10.5281/zenodo.20641515.

## 1 Introduction

Bioimaging is a dynamic field producing quality imaging datasets, some of which have been made publicly accessible as part of a drive to make the data Findable, Accessible, Interoperable and Reusable (FAIR) (Wilkinson *et al.* 2016). Public image data resources are now available to facilitate the access but finding quality data quickly and efficiently remains a challenge, and this in turn limits their reuse and reduces the chance of new discoveries.

In 2016, the Open Microscopy Environment (OME) began a collaboration with EMBL-EBI to build the Image Data Resource (IDR) (Williams *et al.* 2017), an added-value, journal-independent resource, publishing reference bioimage datasets associated with peer-reviewed publications. IDR uses, as its basis, Bio-Formats (Linkert *et al.* 2010) and OMERO (OME-Remote Objects) (Allan *et al.* 2012). Bio-Formats is a suite of libraries which are used heavily by the Java image processing community for reading proprietary scientific image data and metadata into a common model. OMERO is a well-established, secure client-server software platform for image data management and analysis. IDR uses the OMERO database to store image metadata and a customization of the OMERO Web User Interface to publish and explore the data and associated metadata.

Since the creation of IDR, other public bioimage data resources have emerged to collect and share data, including analytical results such as the BioImage Archive (BIA) (Hartley *et al.* 2022) and SSBD (Systems Science of Biological Dynamics) (Tohsato *et al.* 2016).

Together, these resources are now hosting over one petabyte of well-annotated, organised, public bioimage data. When these resources were initially built, the emphasis was placed on data access either via User Interface (UI) or Application Programming Interface (API), to achieve some of the goals formulated in the FAIR principles, specifically Accessible (A) and Interoperable (I) data.

For each study in IDR, image data is stored along with structured and unstructured metadata related to the experimental design, data acquisition, and analysis. OMERO’s database has specific tables for structured acquisition metadata, whereas OMERO flexible key-value annotations are used to store the less structured experimental metadata. This flexible framework is currently used to store, at the image or study level, some of the recommended REMBI metadata (Recommended Metadata for Biological Images) (Sarkans *et al.* 2021) (e.g. organism, study type, imaging method).

Metadata standards and ontologies currently vary between public bioimage data resources. Plus, not all metadata (e.g. structured metadata) is readily indexed and available to standard searchers (Google, Bing, etc.). The combination of these challenges means that datasets which may reveal key insights into biological mechanisms and diseases cannot be easily found, thus limiting data sharing, impact, and reuse.

Over two decades ago, open-source libraries offering full-text search and indexing of data emerged, with Apache Lucene (https://lucene.apache.org/) being one of the flagship tools. These libraries quickly became the backbone of several enterprise search servers, which index either structured or unstructured data from different sources associated with a specific repository, such as a company’s data or data from a specific scientific domain. Two heavily adopted open-source options, both built-on Apache Lucene, are Apache Solr (https://solr.apache.org/) and Elasticsearch (https://www.elastic.co/). Apache Solr is the solution behind the search capabilities of the Protein Data Bank (PDB) (Berman *et al.* 2000), and Elasticsearch is used by the Netflix recommendation engine (https://netflixtechblog.com/reverse-searching-netflixs-federated-graph-222ac5d23576) and the eBay large scale search functionality (https://innovation.ebayinc.com/stories/elasticsearch-performance-tuning-practice-at-ebay/).

In this paper, we introduce a data source-agnostic search solution leveraging Elasticsearch to make bioimaging data hosted in public resources easily and quickly findable (“F” in FAIR).

We describe the use of Elasticsearch to meet the indexing and search requirements of metadata-rich bioimaging repositories like IDR.

## 2 IDR searcher

We developed the IDR searcher (https://github.com/ome/omero_search_engine), an open-source search engine that leverages the full-text and lexical search capability of Elasticsearch. The IDR searcher offers a fast, efficient, cost-effective solution to explore large volumes of data stored in different resources. For this reason, we suggest it as a viable, performant, and scalable search solution for the bioimaging community.

The IDR searcher can index public data from multiple sources, while capturing the provenance, to provide a unified search across combined data sources. Currently, the search engine is deployed in production as the searching solution for IDR. We have nonetheless already explored the possibility of searching data across multiple sources, hosted in different storages. This is a long-standing challenge which is known to be burdened by incompatibility, schema inconsistency, contradictory results, and problems derived from loss of context. The IDR searcher can also be used to search data in any OMERO server hosting public data. Indexing private data is an obvious next step, but such a feature is not yet supported.

### 2.1 Architecture

**Figure 1:**
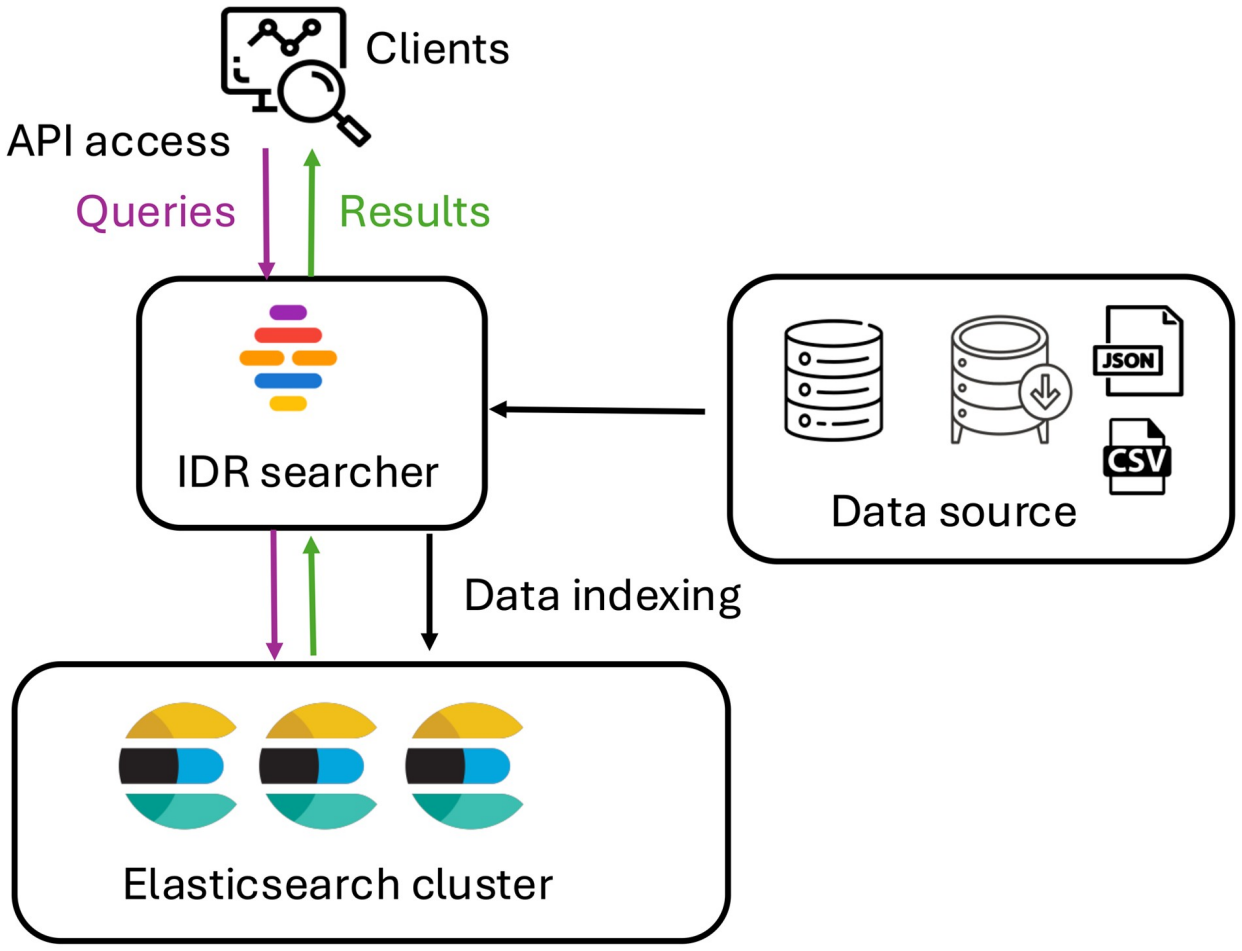
Schematic view of the IDR searcher architecture. Data to be indexed can be retrieved from different sources (e.g. relational databases or Comma-Separated Values (CSV) files). The data is prepared by the IDR searcher and transferred to Elasticsearch for indexing. Users access the indexed data through the IDR searcher API; User’s search requests are translated into Elasticsearch queries. The results returned by Elasticsearch are then reformatted into a simpler structure, making them easier for users and client applications to interpret. *Some icons used to prepare this figure are made by Pixel perfect from*  www.flaticon.com.

The IDR searcher is composed of three components described in the schematic architecture diagram (Fig. 1).

- *Data indexing*: The search engine is independent of the underlying source of data. It can index data stored in diverse structures, such as relational databases, database dumps, or text-based files. The data indexing process is handled through an automated pipeline that extracts and transforms the data before transferring it to Elasticsearch for final storage.
- *API access*: The search engine is easy to use for developers and technically oriented users as it offers a REST (REpresentational State Transfer) API, which is centered around JSON, the widely used and standard format for transferring data.
- *Horizontal scaling*: The search engine is designed to work with several Elasticsearch nodes to guarantee high availability and performance. In this context, horizontal scaling refers to increasing the number of Elasticsearch instances, so the workload is distributed across multiple nodes.

The IDR searcher acts as a bridge between users, data sources, and Elasticsearch. It extracts, prepares, and submits data from various data sources and transfers it to Elasticsearch (Data indexing). It also builds search queries from client requests, submits them to Elasticsearch, and formats the results so they are easy for users or systems to use (see Fig. 1).

### 2.2 Data indexing and caching

The IDR searcher depends on two initial key steps: *data indexing* and *creation of a cache*. The data indexing phase involves systematic retrieval, transformation, and storage of the data in a format optimized for fast, full-text searching. The indexing process works as follows:

- *Data Retrieval*: The operation begins with retrieving the data from its primary source. The search engine is designed to be data-source agnostic. Possible data sources include relational databases or text files.
- *Data Ingestion* **:** Once retrieved, the search engine ingests the data into Elasticsearch for search and retrieval. This process involves:
  - *Data Transformation and Formatting* **:** The data is transformed into structured JSON documents based on a predefined Elasticsearch template which describes how fields should be indexed and searched.
  - *Data Push to Elasticsearch* **:** The formatted data is then pushed to Elasticsearch via its REST API, where it is processed to a form that enables fast searching.

The cache data refers to aggregated and summarized information generated from the indexed records to provide high-level insights and improve data findability and discoverability. The cache is systematically built once the entire indexing process has been successfully completed. This ensures that the summary contained in the cache accurately reflects the full, up-to-date state of the indexed data. Key information, including the total datasets’ count and statistics on the main experimental parameters, is stored in the cache. The cache, for example, provides an immediate overview of the number of images linked to specific organisms. These statistics allow the user interface to display accurate image counts next to organism names in a list of search results. The users can then quickly gauge the volume of data available for a particular study subject without executing a complex query.

**Figure 2:**
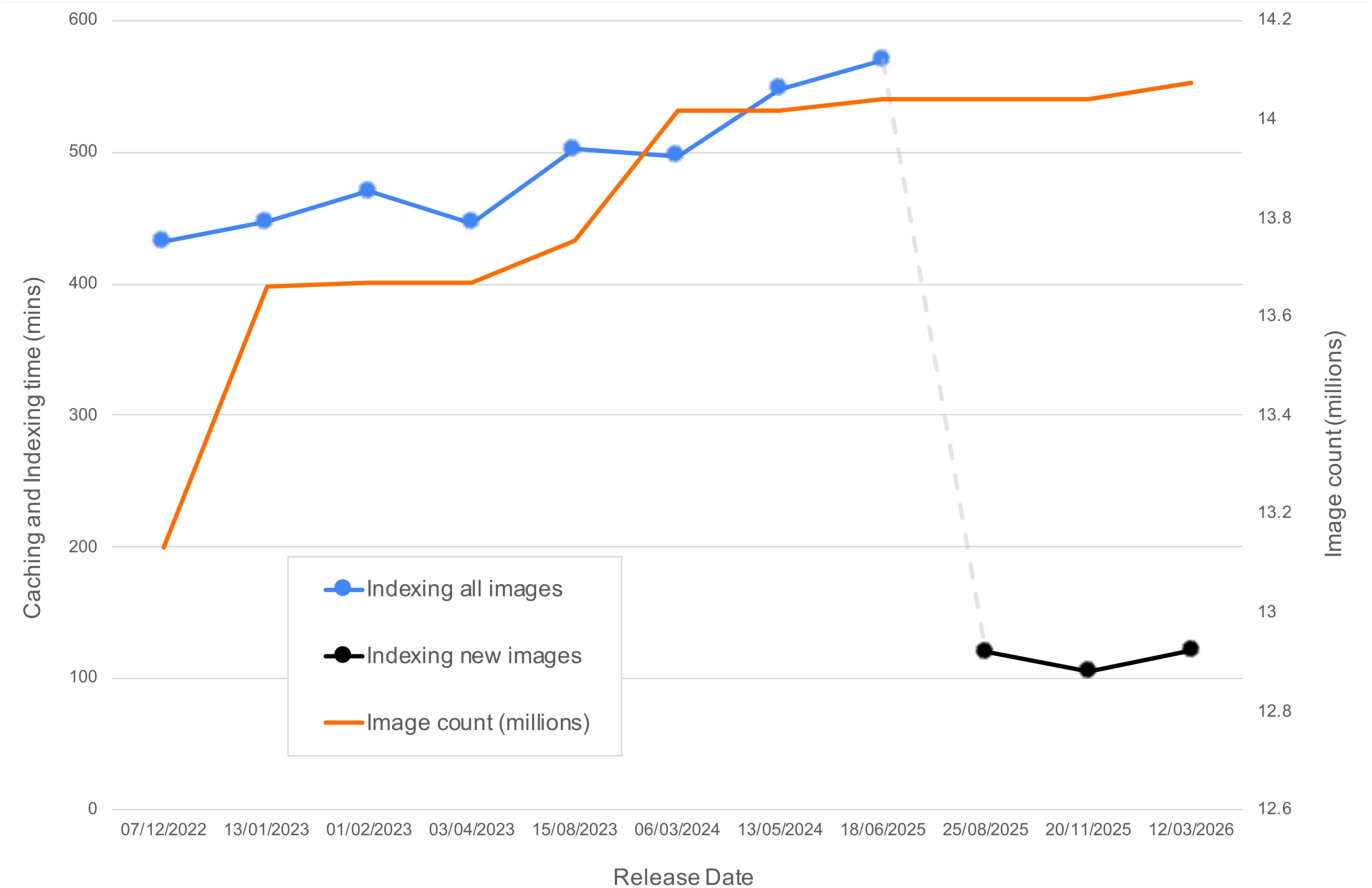
The graph shows the total time required for indexing and caching publication releases within IDR. The total image count was 13.1 million on 7 December 2022, increasing to 14 million by 25 August 2025. The rise in total image number in IDR (orange curve, number of images [millions]) and the indexing and caching time (blue curve, time [minutes]) were clearly correlated. The new indexing strategy was introduced between 18 June 2025 and 25 August 2025 (light-grey dashed line). Since 25 August 2025, only the metadata from newly added images was indexed for each release (black curve, time [minutes]). This dramatically shortened indexing and caching times (from 570 min in June to 120 min in August, the last point of the blue curve and the first point of the black curve, respectively).

Data is being continuously added to bioimaging resources. For IDR, this was leading to a significant increase in the time required to index the metadata and update the cache; steps that needed to be run before each IDR release. This increasing overhead would eventually compromise the time-schedule for publishing new datasets in IDR. To address this issue, we focused on reducing the indexing workload (see Fig. 2). We enhanced the search engine to index only newly added or updated data and metadata and reuse the existing index for the rest of the hosted data and metadata. This improvement, implemented first for the IDR release on 25 August 2025, reduced the pre-release indexing and caching time by approximately seven hours (from 570 min to 150 min total time for indexing and caching) in comparison with the previous release on 18 June 2025. The indexing time is solely related to the volume of metadata associated with the images and not to the size or resolution of the images. The cache needs to be rebuilt for all images prior to each release. We also cannot measure the caching and indexing times separately. Thus, the observed shortening of the indexing and caching times are because we indexed fewer images after the release on 25 August 2025.

We aim to refine our current caching strategy, which should lead to further time reductions.

### 2.3 API access: searching the data

The IDR searcher API can be accessed via HTTP requests to a number of well documented URLs. The response data is provided in the form of JSON (https://idr.openmicroscopy.org/searchengine/apidocs/), allowing other applications to access the indexed data easily.

We have prepared a collection of notebooks (https://workflowhub.eu/collections/36) hosted in the WorkflowHub (Gustafsson *et al.* 2025), demonstrating how to use the search engine API to explore the richness of IDR.

### 2.4 Deployment

To manage the deployment of the IDR searcher, we use Ansible (https://docs.ansible.com/), an automation tool to configure, manage and deploy applications. The Ansible specifications for deploying IDR searcher are open-source and publicly available (https://github.com/ome/ansible-role-omero-searchengine). The instance of the search engine deployed alongside IDR is managed by Ansible on an OpenStack-based cloud contained within the EMBL-EBI Embassy resource. It is configured to use three Elasticsearch nodes deployed on the same Virtual Machine to ensure high availability.

### 2.5 Use cases

#### 2.5.1 A performant search engine for IDR

When IDR was first created, we initially developed and used OMERO.mapr (https://github.com/ome/omero-mapr) as the search solution based on direct queries of key-value pairs stored in the IDR relational database. As the volume of metadata increased, the time needed for any direct search query to the database using OMERO.mapr increased significantly.

To compare performance and ensure that equivalent queries using either tool returned identical results, we deployed both OMERO.mapr and IDR searcher side-by-side. We used a single Elasticsearch node to ensure a fair performance comparison between applications. We performed several different queries that returned different numbers of images, searching for a specific Gene Symbol, Compound Name, or Phenotype (e.g. Gene Symbol = pak1 or Compound Name = antimycin a). OMERO.mapr, even for simple queries, requires combining information from multiple tables in the relational database to return the relevant values. As the number of key-value pairs increases, the size of the tables increases.

**Figure 3:**
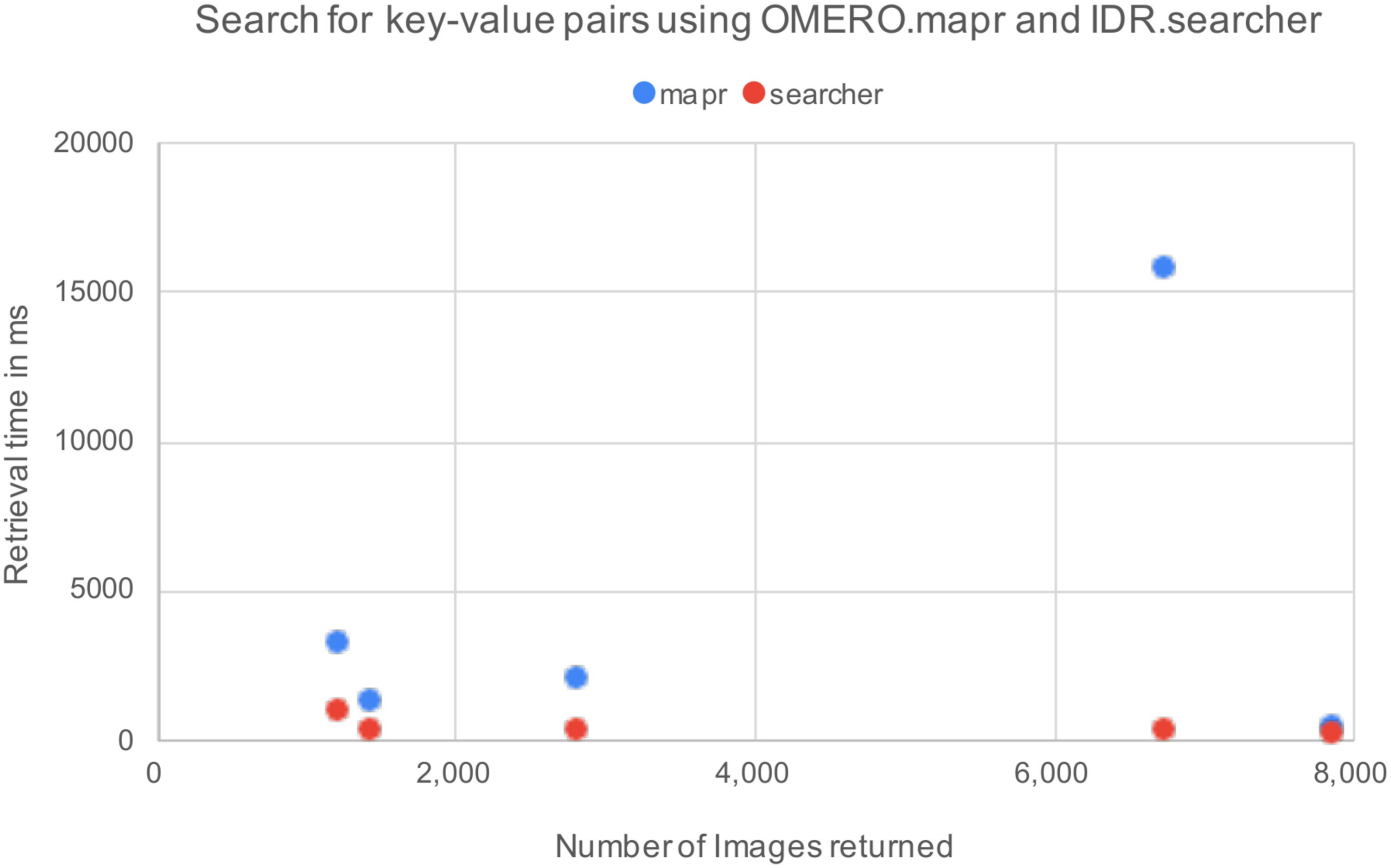
Scatter plot comparing the time to perform five different queries using both OMERO.mapr and IDR searcher. For each query, the same images are returned using either application.

Figure 3 highlights a performance improvement for each query. OMERO.mapr query times are highly variable depending on the query complexity, but in general are two to five times slower than the equivalent query using the IDR searcher. In particular, the search for Gene Symbol = incenp, was significantly slower in OMERO.mapr (15.8 seconds), compared to 0.34 seconds in IDR searcher. This is because in the IDR searcher, the results are retrieved through the cache instead of a direct query to tables in the database. The performance of the IDR searcher can easily be improved by adding more Elasticsearch nodes to the search cluster.

#### 2.5.2 User interfaces to the IDR searcher

**Figure 4:**
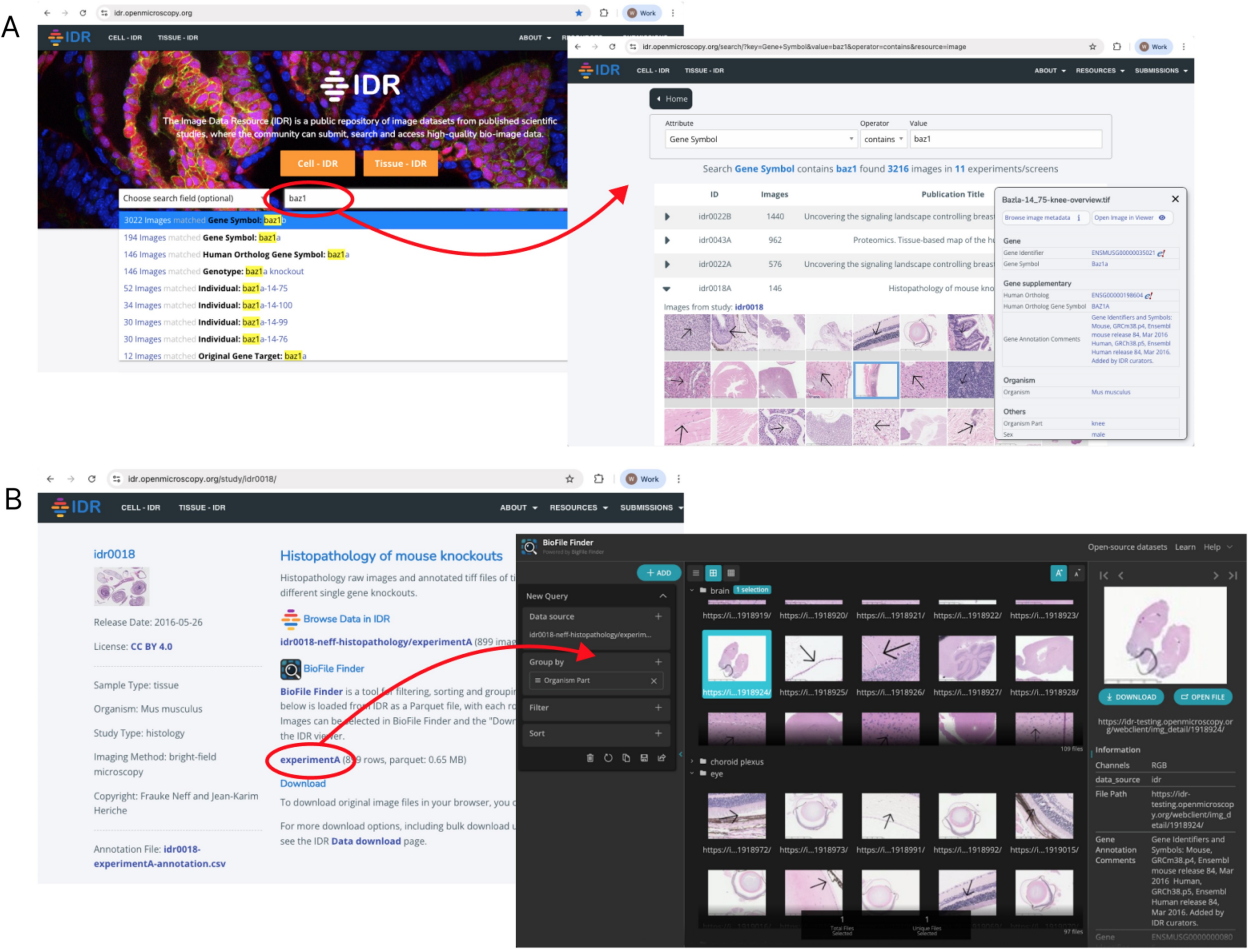
(A) Auto-complete terms are retrieved from the search engine as the user types in a query. Search results are displayed on a new page where they can be explored in detail. (B) Study pages in IDR include links to open all images from that study in BioFile Finder (BFF). Tabular data is loaded into BFF using the IDR searcher in the form of Parquet files. The table includes links to thumbnails, which are then directly loaded from the IDR.

To enable web-based users of IDR to access the search engine, we have adapted the IDR User Interface to work with the search engine API. As users enter search terms, the search engine is used to find auto-complete matches from across the entire search index, ensuring that no terms of interest are missed. On selection of a search key and value, a full search is performed for matching images, which are displayed on the results page (see Fig. 4A). This page allows the user to refine their search and inspect all Key-Value metadata of retrieved images. The Key-Value pairs are displayed as links that will execute further searches for Images with identical attributes. By following such links, a user is able to browse and explore the full search engine index. A video demonstrating how to use the search engine from IDR User Interface is available on the IDR website (https://idr.openmicroscopy.org/).

##### 2.5.2.1 Integration with BioFile finder

Studies in added-value resources like IDR usually have large amounts of associated metadata requiring applications whose primary focus is metadata exploration. BioFile Finder (https://bff.allencell.org/) is a Web application from the Allen Institute for Cell Science designed for easy access and sharing of images through metadata search, filtering and sorting. We added to the IDR searcher the ability to export the indexed metadata associated with a study to CSV files or to tables stored in the optimized and compressed Apache Parquet format. Those files can be directly opened in BioFile Finder to offer the user a new way to visually explore, sort, and filter the data and metadata stored in IDR (see Fig. 4B). Example CSV files can be found in the IDR searcher GitHub repository (https://github.com/ome/omero_search_engine/blob/main/README.rst#Key-Features).

This example illustrates the value of bioimage data sharing and shows how the IDR searcher has the potential to contribute to a broader shift toward a more open research culture in bioimaging.

### 2.6 Training and dissemination

The IDR searcher guide is available online (https://omero-search-engine.readthedocs.io/en/latest/index.html), providing a range of resources to support both administrators and users. The application was presented during recent Imaging workshops and courses (https://www.ebi.ac.uk/training/materials/microscopy-data-analysis-machine-learning-and-the-bioimage-archive-2026/) focusing on the exploration of public repositories. A proposed training plan is outlined in the IDR searcher guide (https://omero-search-engine.readthedocs.io/en/latest/training_plan/training_plan.html). A detailed training plan, along with the supporting training materials, will be delivered upon request. Information about the application has been published on the image.sc forum (https://forum.image.sc/) and will continue to engage with the community.

## 3 Conclusions

We have developed the IDR searcher to provide an efficient solution for searching through a large volume of public bioimage metadata, which continues to grow exponentially. The performance of the application indicates its potential as a valid alternative to the search engine currently included in OMERO. The current version of IDR searcher can be used to index publicly accessible metadata in any OMERO instance. We are currently working on interfacing it with the OMERO permissions system so it could also be used with any OMERO installation which is protected by the OMERO authentication and permission system.

The IDR searcher application not only helps to find public bioimage data efficiently but also allows the exchange of metadata between public resources like IDR and tailored metadata visualization tools like BioFile Finder.

The development of OME-Zarr, a cloud-optimized image format (Moore *et al.* 2021) has greatly simplified the creation of public resources, allowing many institutions to make their data accessible (https://ngff.openmicroscopy.org/resources/data/index.html). However, the re-use of the image data is limited by the lack of search across decentralized repositories. The IDR searcher has been designed with this use-case in mind, and we are already testing the indexing and search of OME-Zarr images. Therefore, the IDR searcher could provide the foundation for searching, via a single index, through public centralized repositories like IDR or SSBD and decentralized repositories hosting OME-Zarr data.

We would also like to explore the use of RO-crate (Soiland-Reyes *et al.* 2022) as a format for output of search results from the IDR searcher. This would support the preservation of image and metadata in a format that can be understood and cited by other tools.

This ongoing work on the IDR searcher will improve findability and access to quality bioimage data, promoting its reuse across the bio-imaging community.

## Data Availability

No new data were generated or analysed in support of this research.
